# Behavioral and neuro-functional consequences of eliminating the KCNQ3 GABA binding site in mice

**DOI:** 10.3389/fnmol.2023.1192628

**Published:** 2023-05-25

**Authors:** Kiki J. Chen, Ryan Yoshimura, Clarissa Adriana Edmundo, Tri Minh Truong, Olivier Civelli, Amal Alachkar, Geoffrey W. Abbott

**Affiliations:** ^1^Department of Pharmaceutical Sciences, School of Pharmacy and Pharmaceutical Sciences, University of California, Irvine, Irvine, CA, United States; ^2^Bioelectricity Laboratory, Department of Physiology and Biophysics, School of Medicine, University of California, Irvine, Irvine, CA, United States; ^3^UC Irvine Center for the Neurobiology of Learning and Memory, University of California, Irvine, Irvine, CA, United States; ^4^Institute for Genomics and Bioinformatics, School of Information and Computer Sciences, University of California, Irvine, Irvine, CA, United States

**Keywords:** KCNQ3, GABA, binding, behaviors, mutation

## Abstract

Voltage-gated potassium (Kv) channels formed by α subunits KCNQ2-5 are important in regulating neuronal excitability. We previously found that GABA directly binds to and activates channels containing KCNQ3, challenging the traditional understanding of inhibitory neurotransmission. To investigate the functional significance and behavioral role of this direct interaction, mice with a mutated KCNQ3 GABA binding site (Kcnq3-W266L) were generated and subjected to behavioral studies. Kcnq3-W266L mice exhibited distinctive behavioral phenotypes, of which reduced nociceptive and stress responses were profound and sex-specific. In female Kcnq3-W266L mice, the phenotype was shifted towards more nociceptive effects, while in male Kcnq3-W266L mice, it was shifted towards the stress response. In addition, female Kcnq3-W266L mice exhibited lower motor activity and reduced working spatial memory. The neuronal activity in the lateral habenula and visual cortex was altered in the female Kcnq3-W266L mice, suggesting that GABAergic activation of KCNQ3 in these regions may play a role in the regulation of the responses. Given the known overlap between the nociceptive and stress brain circuits, our data provide new insights into a sex-dependent role of KCNQ3 in regulating neural circuits involved in nociception and stress, *via* its GABA binding site. These findings identify new targets for effective treatments for neurological and psychiatric conditions such as pain and anxiety.

## Introduction

The muscarinic-inhibited current (M-current) is a highly influential voltage-gated K^+^ (Kv) current in the central nervous system (Brown and Adams, 1980; Wang et al., 1998). Generated by KCNQ (Kv7) family Kv channels, primarily heteromeric and perhaps homomeric channels containing KCNQ2, 3 and/or 5, M-current plays a vital role in regulating the membrane potential and excitability of neurons. KCNQ2/3 channels in particular are located at sites of action potential initiation and regeneration, making them essential regulators of neuronal function (Cooper, 2011).

Given the importance of M-current in controlling neuronal excitability, neuronal KCNQ channels have emerged as promising targets for the treatment of various disorders related to hyperexcitability. These include conditions such as acute and neuropathic pain, migraine pain, anxiety, epilepsy, stroke, and traumatic brain injury (Singh et al., 1998; Wua and Dworetzky, 2005; Munro and Dalby-Brown, 2007; Bierbower et al., 2015; Li et al., 2019; Vigil et al., 2020; Zhou et al., 2022). The inhibitory effects of presynaptic KCNQ2/3 channels on neuronal excitability have been partially attributed to their modulation of the release of GABA, the primary inhibitory neurotransmitter in the central nervous system (Martire et al., 2004; Peretz et al., 2007).

**Figure 1:**
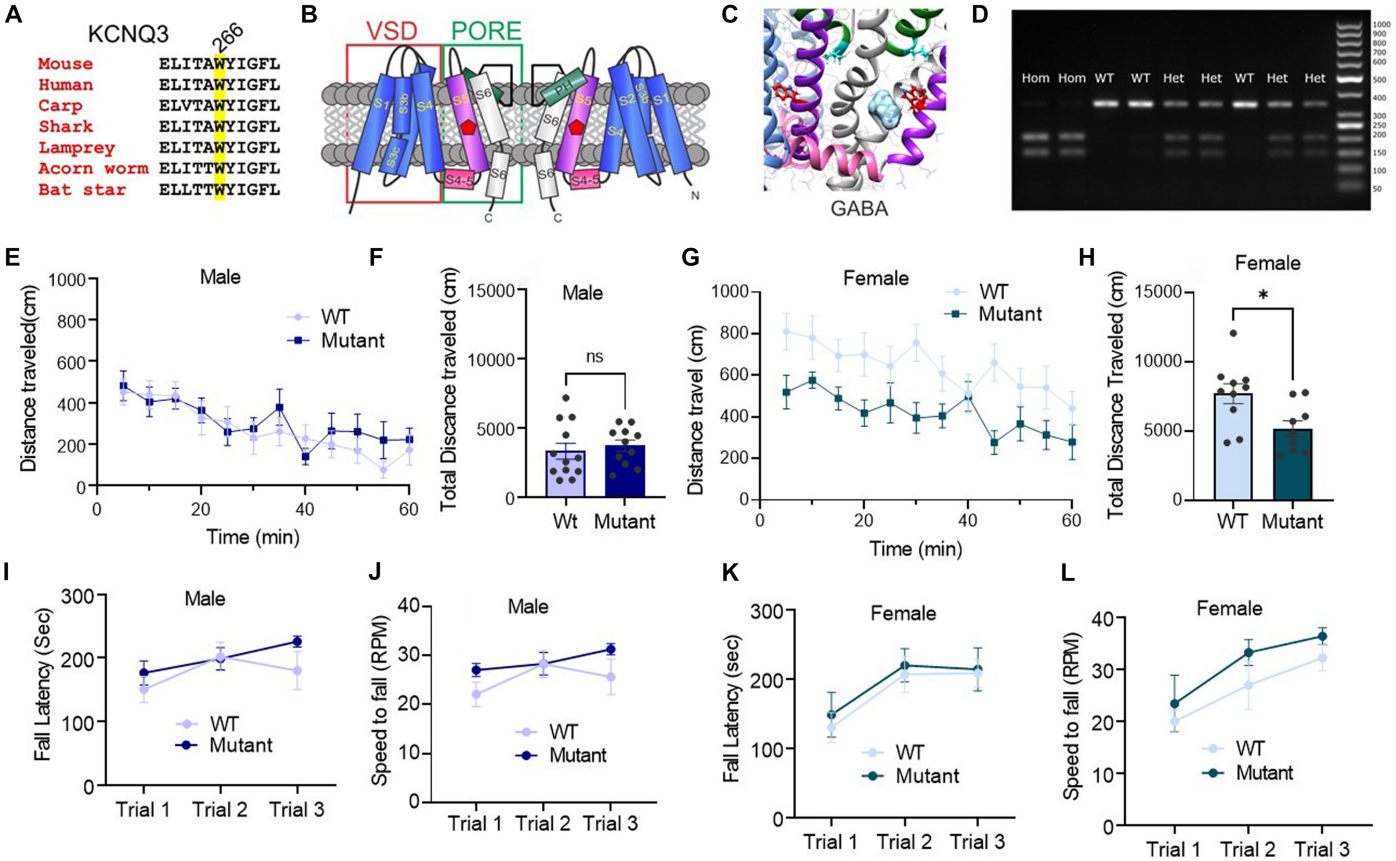
Germline disruption of the KCNQ3 GABA binding site does not affect motor coordination, but it affects female spontaneous motor activity. **(A)** Sequence alignment of part of KCNQ3 transmembrane segment S5 showing conservation of W266 (highlighted; mouse numbering). **(B)** Cartoon showing two subunits of KCNQ3 and approximate location of W266 (green circle). VSD, voltage sensing domain. **(C)** Image of SwissDock *in silico* docking prediction of GABA to the S5 W (red) (adapted with permission from Manville et al. (2018)). **(D)** Representative PCR genotyping gel of DNA samples from mice in the Kcnq3-W266L mouse colony to confirm the presence or absence of the W266L mutation. Hom, homozygous W266L; Het, heterozygous W266L; WT, wild-type. Right-hand lane: molecular weight markers (numbers indicate base pairs). **(E–H)** Spontaneous motor activity: **(E)** Male: distance traveled every 5 min in the last 60 min of the locomotion assay (*n* = 12 WT, 11 Kcnq3-W266L); **(F)** Male: total distance traveled over 60 min. Unpaired t-test (*t* = 0.5455, *p* = 0.5912), WT vs. Kcnq3-W266L, ns, not significant. **(G)** Female: distance traveled every 5 min in the last 60 min of the locomotion assay (*n* = 10 WT, 10 Kcnq3-W266L); **(H)** Female: total distance traveled over 60 min. Unpaired t-test (*t* = 2.679, *p* = 0.0158), WT vs. Kcnq3-W266L, ^*^p < 0.05, Data are presented as mean ± s.e.m. **(I–L)** Motor coordination on accelerating rotarod. **(I)** Latency to fall in rotarod assay (male WT *n* = 8, Kcnq3-W266L *n* = 10). Two-way ANOVA (genotype effect: *F* (1,48) = 1.959, *p* = 0.1680, Trial effect: *F* (2,48) = 2.422, *p* = 0.0995), followed by Bonferroni *post hoc* test: Kcnq3-W266L vs. WT, ns, not significant; **(J)** Speed to fall in rotarod assay (male WT *n* = 8, Kcnq3-W266L *n* = 10). Two-way ANOVA (genotype effect: *F* (1,48) = 1.928, *p* = 0.1714, Trial effect: *F* (2,48) = 2.510, *p* = 0.0919). Followed by Bonferroni *post hoc* test: Kcnq3-W266L vs. WT, ns, not significant; **(K)** Latency to fall in rotarod assay (female WT *n* = 9, Kcnq3-W266L *n* = 10). Two-way ANOVA (genotype effect: *F* (1,51) = 0.3159, *p* = 0.5765, Trial effect: *F* (2,51) = 4.962, *p* = 0.0107), followed by Bonferroni *post hoc* test: Kcnq3-W266L vs. WT, ns, not significant; **(L)** Speed to fall in rotarod assay (female WT *n* = 9, Kcnq3-W266L *n* = 10). Two-way ANOVA (genotype effect: *F* (1,27) = 2.619, *p* = 0.1172, Trial effect: *F* (2,27) = 6.708, *p* = 0.0043). Data are presented as mean ± S.E.M.

Our recent research unexpectedly uncovered that GABA can bind to KCNQ3 and KCNQ5-containing channels, with affinities similar to those of canonical GABAA receptors, causing activation of KCNQ3 and KCNQ5 and consequent cellular hyperpolarization (Manville et al., 2018). GABA binding to KCNQ3 or KCNQ5 requires a highly conserved Trp (W) residue on the S5 transmembrane segment (W265 in human KCNQ3; W266 in mouse Kcnq3) (Figures 1A–C) (Manville et al., 2018). This direct activation of KCNQ channels by GABA indicates that there may be other mechanisms underlying inhibitory GABA-neurotransmission signaling and raises important questions about the physiological and pathological implications of this interaction.

The direct activation of KCNQ channels by GABA challenges the traditional understanding of its inhibitory neurotransmission in the central nervous system. It was traditionally believed that the inhibitory effects of GABA on neuronal excitability are mediated solely through its classical receptors: (1) GABA-A, ligand-gated ion-channels, which mediate GABA fast inhibitory transmission, through chloride conductance that suppress neuronal excitability and (2) GABA-B receptors, G-protein-coupled receptors (GPCRs), which mediate GABA slow inhibitory signaling through activating other proteins, including specific non-KCNQ-K^+^ channels (Cooper et al., 2001; Tzingounis et al., 2010). The discovery of the direct activation of KCNQ channels by GABA has opened up new avenues for exploring other, previously unknown, mechanisms underlying inhibitory GABA-neurotransmission signaling in the central nervous system. The physiological implications of this direct interaction between GABA and KCNQ channels are as yet unknown. The direct activation of KCNQ channels by GABA also raises questions about a potential role in pathological conditions and suggests new targets for the development of effective treatments for these conditions.

To begin to explore the functional significance and behavioral role of the direct interaction between GABA and KCNQ channels, here, using CRISPR technology, we generated mice with a mutation (Kcnq3-W266L) that we previously found *in vitro* to prevent GABA binding to and activation of KCNQ3 (human equivalent, KCNQ3-W265L) (Manville et al., 2018). We then conducted a series of behavioral studies on male and female Kcnq3-W266L mice. In addition to the behavioral studies, we also used a cFos immunoreactivity assay to study the changes in neuronal activity associated with mutation of the KCNQ3 GABA binding site.

## Materials and methods

### Animals

All experimental procedures were approved by the Institutional Animal Care and Use Committee of the University of California, Irvine, and procedures were performed following national and institutional guidelines for the care and use of laboratory animals.

### Generation of Kcnq3-W266L mice using CRISPR/Cas9 modification

Knock-in C57BL6/NJ mice with a W266L mutation in Kcnq3 (chromosome 15, NCBI sequence NC_000081.7) were generated by the UC Irvine Transgenic Mouse Facility using CRISPR/Cas9 modification. Guide RNA (g226 TCTAGGAACTCATCACTGCC and g227 TGTCAGGAAGCCTATGTACC), tracrRNA, Cas9 protein, and the repair template (ssODN TMF1128 CCTGCTGACTCCCTCTGTT GTTCACTGTTCTAGGAACTCA TCACTGCtttgTACATAGGCTTC CTGACACTCATCCTTTCTTC ATTTCTTGTCTACCTGGTGG) were prepared by Integrated DNA Technologies (Coralville, IA, United States). Pronuclei from C57BL6/NJ oocytes were injected with the ribonucleoprotein complex and repair template. Surviving embryos were implanted into ICR foster mothers. A total of 6 pups survived without insertion/deletion mutations, of which 4 potentially had the correct sequence. Two mice passed the desired sequence through the germline and were used as founders for the transgenic colony.

KCNQ3-W266L mice were set up in heterozygous-heterozygous breeding pairs to generate wild-type, heterozygous, and homozygous mutant mice for testing. DNA from offspring mice was isolated from a tissue sample for genotyping. PCR was performed on each DNA sample (5’-GGATAGGAGGTGAGACTCAGAAAAG-3′ and 5’-CTTCCCTCTGCATCTAGTGGTCTC-3′, 343 nt) and the resulting PCR product was then digested with BsrGI (New England Biolabs, Ipswich, MA, United States) to confirm the presence or absence of the W266L mutation (Figure 1D).

### Behavioral experiments

Mice were tested with multiple behavioral experiments. They followed the experimental paradigms with the following order: spontaneous locomotion activity and Open Field (OF), T-maze spontaneous alternation, elevated-plus maze, social interaction and novelty, self-grooming, novel object recognition (NOR), hotplate assay, pre-pulse inhibition (PPI), forced swim test (FST), and contextual fear conditioning (CFC). A 4–6 day inter-assay interval was placed among the sequential specific assays. Animals were placed in the test room for each assay 30 min before the experiment for acclimation.

### Open field and spontaneous locomotor activity test

*Animals were placed in a* 40x40x40 cm locomotion chamber box for a total of 90 min (Alachkar et al., 2018). The first 30 min is the habituation time to the chamber box for the animals. The first 10 min of the habituation time was used to access the level of anxiety when the mice were placed in a novel environment and an open area. Total distance, total time spent, the distance in the central square and peripheral area, and the time spent in the two areas were analyzed using Activity Monitor 5 (Med Associates, Inc.). The percent time spent in the center was calculated by using equation, time oF(ambulation in the center zone/time ambulation in moving)x100. The percent time spent at the peripheral area was calculated by using equation, time oF(ambulation in the peripheral area/time ambulation in moving) x100. The spontaneous locomotor activity of the animals was logged every 5 min, over 60 min after the first 30 min habituation and analyzed using Activity Monitor 5 software (Med Associates, Inc.).

### T-maze spontaneous alternation test

Mice were placed and restricted at the base of the T-maze for 30 s for acclimation (Chen et al., 2020). The door was opened for the animals to freely explore either the left or right of the maze after the acclimation. The choice of the left or right arm was recorded. Mice were allowed to explore the chosen arm for 30 s and then returned to the base of the maze to start the subsequent trial. There were 7 possible alternations in total eight trails. The percent alternation was calculated as the equation, (number of alternations/7) x 100. The latency of decision-making was also recorded.

### Elevated-plus maze

The apparatus has two open arms and two closed arms with non-transparent walls and was placed 50 cm above the floor. This assay was conducting in a dark room with two desk lamps shining to the two open arms. Mice were placed in the center of the maze and free to explore either the open or close arm for 5 min. The amount of time spent with the head and forepaws in the different arm and the number of entrances were recorded using ANY-MAZE software (Stoelting, Wood Dale, IL, United States).

### Social interaction and social novelty

The social interaction and social novelty assays were conducted using a rectangular 3-chamber box with removable doors and mesh wire cups in the left and right chambers (Alhassen et al., 2021). The social interaction assay began with a 5-min acclimation period for the mouse in the middle chamber. Then, a stranger mouse of the same sex, age, and strain was placed in one of the mesh wire cups, while the other cup remained empty. The doors were opened, and the experimental mouse was allowed to explore all 3 chambers for 10 min, while the time spent interacting with both the stranger mouse and the empty cup was recorded.

The social novelty assay was conducted immediately after the social interaction assay, where a new stranger mouse with the same sex, age, and strain was placed under the empty cup, while the previous stranger mouse remained in the other chamber. The experimental mouse was returned to the middle chamber with the doors closed and then allowed to explore the box again for 10 min. The time spent interacting with the two stranger mice was recorded, using ANY-MAZE (Stoelting, Wood Dale, IL, United States) software for analysis.

### Self-grooming behavioral assay

Mice were placed in an empty cage for a total of 20 min (Sanathara et al., 2018). After 10 min’ acclimation in the cage, the grooming activity was monitored. The time spent on grooming was recorded manually for 10 min.

### Novel object recognition assay

There are two phases in the novel object recognition (NOR) assay, a training phase and a testing phase (Vawter et al., 2020). All animals were handled 2 min a day prior to the training session and were placed in an empty rectangle experimental apparatus for 5 min for a consecutive 3 days for habituation. During the training session, two identical objects were placed in the apparatus, and mice were allowed to explore the objects for 10 min. The test phase was conducted 24 h after the training session. Each mouse was placed in the apparatus with one familiar and one novel object for 10 min. The total time mice spent interacting with both the familiar and novel objects were recorded using ANY-MAZE software (Stoelting, Wood Dale, IL, United States).

### Hot plate

Mice were placed on the hot plate apparatus without heat for 2 min, 24 h prior to the test. Following the habituation, animals were placed on a 52°C hot plate and monitored for an initial response of either a rear paw lift, paw shake, or paw lick in response to the heat. The time, once the initial response was seen, was recorded. Mice were returned to their home cage. There was a total of 4 trials with 15 min intertrial interval. The average responding time was analyzed.

### Rotarod test

Animals were placed on an elevated rotating rod divided into 5 lanes with trip plates below each lane to sense the falling of the subject (Alhassen et al., 2023). Each trial was 300 s with a starting speed at 4 RPM up to 40 RPM. The fall latency and speed were recorded by Rod (TSE system, Chesterfield, United States). Individual mouse experiments were manually stopped if the animal was unable to run on the rod but rotated with the rod. There were 3 trials with 15 min intertrial interval for each mouse.

### Pre-pulse inhibition

Mice were habituated to the startle chambers for 5 min with 65 dB of background noise 24 h prior to the experiment (Chen et al., 2020). The pre-pulse inhibition sessions consist of 5 trials with either one of the 3 pre-pulse (20-millisecond duration pre-pulse at 68 dB, 71 dB, or 70 dB with a 100-millisecond interstimulus interval to startle stimulus) followed by a 40-millisecond duration startle stimulus at 120 dB. The startle response was recorded using startle response system (San Diego Instruments. San Diego, United States). The amount of pre-pulse inhibition is calculated as a percentage using the equation: % PPI = 100-([(startle response for pre-pulse + pulse trials)/ (startle response for pulse-alone trials)] ^*^ 100).

### Forced swim test

Mice were placed in a cylinder contained with 25°C water for a total of 6 min (Alhassen et al., 2021). After the first 2 min adaptation to the water, the immobility time or the time the mice spent floating was recorded using Any-maze software (Stoelting, Wood Dale, IL, United States).

### Contextual fear conditioning

This assay consisted of two sessions: a training session and a testing session, which was conducted 24 h after the training session (Alachkar et al., 2018). During the training session, mice were placed in a conditioning chamber for 3 min, during which time they received a 2-s 0.7 mA foot shock at 2.5 min. The mice were then returned to their home cage. During the testing session, mice were returned to the same chamber for 5 min without shock. The freezing behavior of the mice was measured for pre- and post-shock training and testing sessions. Freezing behavior was recorded as either “1” (if freezing occurred within a 5-s interval) or “0” (if no freezing occurred within a 5-s interval). The percentage of freezing behavior was calculated as 100*(number of intervals of freezing/total intervals).

### Immunohistochemistry

Mice were anesthetized with isoflurane and transcardially perfused with 0.9% saline followed by 4% paraformaldehyde (PFA) at 90 min after the contextual fear conditioning. Harvested brains were kept in 4% PFA overnight and then transferred to 30% sucrose the next day for cryoprotection. Brains were coronally sectioned at 30 μm using a microtome. One section from the region of interest was selected from both the wildtype (WT) and KCNQ3 homogeneously W266 mutated group. The sections were blocked with donkey serum in PBS with 0.3% Triton X-100 for 1 h. The brain sections were then incubated in blocking buffer with primary antibodies, c-Fos 1:500 (Abcam ab190289 Rb pAb, Lot#: GR339395) and KCNQ3 (Santa Cruz Biotechnology, KCNQ3 sc-7,794 goat pAb to KCNQ3 Lot#: C3114) for 24 h. The sections were washed three times with PBS prior to secondary antibody incubation. The sections were incubated with the secondary antibodies, 1:500 (Sigma Anti-goat IgG (H + L) produced in donkey ref.: SAB4600074 Lot: 16C0420), AlexaFluor488 donkey anti-rabbit 1:500 (Invitrogen, ref.: A21206 Lot: 1275888), and DAPI 1:10000 (Thermo Scientific Ref: 62248 Lot: WF3296471). The sections were then washed with PBS and mounted on slides for imaging. The images were taken using the Keyence BZ-9000 microscope with a 10x objective lens for c-Fos positive neurons and KCNQ3 positive neurons. c-Fos positive neurons were counted in the area selected from left and right sides of the regions of interest. The counts for the left and right hemispheres were combined and averaged, and the cell density was calculated by dividing the total number of c-Fos positive neurons by the area of the selected region. Fiji (ImageJ) was used for image analysis and cell counting.

### Statistical analysis

Statistical analysis was conducted using GraphPad Prism software (GraphPad Software, Inc.). The data were expressed as means ± S.E.M. Statistical significance was determined using Student t-test or ANOVA, with *p* < 0.05 considered as the level of significance in the appropriate *post hoc* comparison.

## Results

### Germline disruption of the Kcnq3 GABA binding site affects spontaneous motor activity in female mice, but does not impact motor coordination

We first analyzed the effects on motor activity of mutating the Kcnq3 GABA binding site (Kcnq3-W266L). Two behavioral assays were used: an open field test to assess spontaneous motor activity, and a rotarod test to evaluate motor coordination.

Our results showed that female Kcnq3-W266L mice had significantly reduced locomotor activity compared to wild-type mice, while male Kcnq3-W266L mice displayed activity levels similar to those of wild-type mice (Figures 1E–H). Results from the rotarod test showed no significant difference in motor coordination between the Kcnq3-W266L and wild-type mice in both males and females (Figures 1I–L). These findings suggest that mutating the KCNQ3 GABA binding site does not significantly affect motor coordination but may impact spontaneous motor activity in female mice.

### Germline disruption of the Kcnq3 GABA binding site does not alter sociability, sensorimotor gating and depression-like behaviors

We next performed social interaction assays to study the effect on sociability, sensorimotor gating, and depression-like behavior.

**Figure 2:**
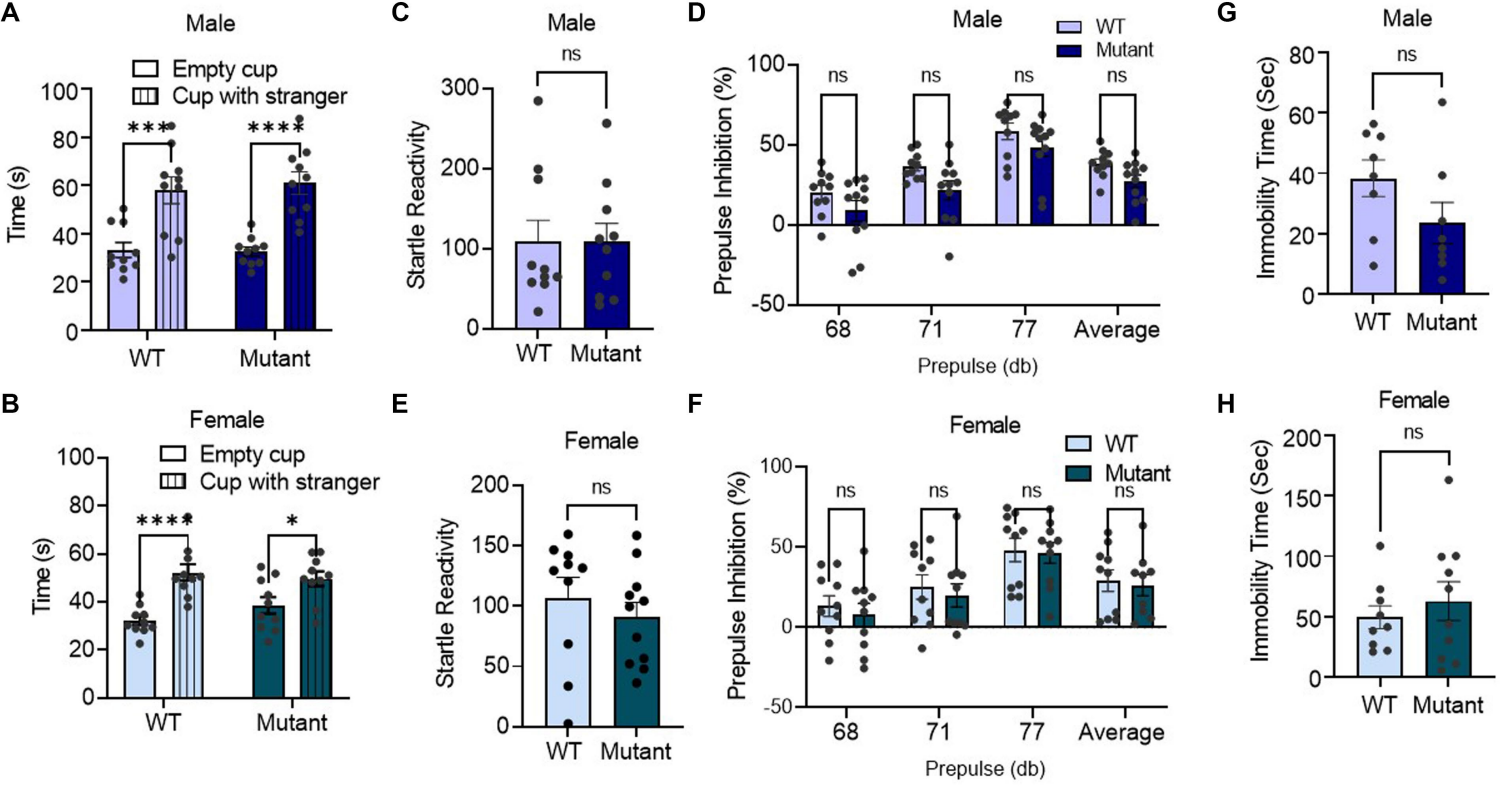
Germline disruption of the KCNQ3 GABA binding site does not affect sociability, sensorimotor-gating behavior, and depression-like behavior. **(A)** Social interaction in male (*n* = 10 WT, *n* = 10 Kcnq3-W266L). Two-way ANOVA (cup effect: *F* (1,36) = 42.96, *p* < 0.0001. genotype effect: *F* (1,36) = 0.07994, *p* = 0.7790); followed by Bonferroni *post hoc* test: Empty cup vs. Stranger Mouse, ^****^p < 0.0001. Data are presented as mean ± S.E.M. **(B)** Social interaction in female (*n* = 10 WT, *n* = 10 Kcnq3-W266L). Two-way ANOVA (cup effect: *F* (1,36) = 27.28, *p* < 0.0001, genotype effect: *F* (1,36) = 0.3962, *p* = 0.5330); followed by Bonferroni *post hoc* test: Empty cup vs. Stranger Mouse, ^****^ *p* < 0.0001. Data are presented as mean ± S.E.M. (c-f) Performance of mice in prepulse inhibition assay. **(C)** Startle reactivity in prepulse inhibition assay in male (*n* = 10 WT, *n* = 10 Kcnq3-W266L), Unpaired t-test (*t* = 0.01024, *p* = 0.9919), ns, not significant. **(D)** Average prepulse inhibition in male (*n* = 10 WT, *n* = 10 Kcnq3-W266L), Two-way ANOVA (genotype effect: *F* (1,76) = 11.92, *p* = 0.0009, prepulse intensity effect: *F* (3,76) = 21.91, *p* < 0.0001), followed by Bonferroni *post hoc* test: Kcnq3-W266L vs. WT, ns, not significant. **(E)** Startle reactivity in prepulse inhibition assay in female (*n* = 10 WT, *n* = 10 Kcnq3-W266L), Unpaired t-test (*t* = 0.7908, *p* = 0.4388), ns, not significant. **(F)** Average prepulse inhibition in female (*n* = 10 WT, *n* = 10 Kcnq3-W266L), Two-way ANOVA (genotype effect: *F* (1,71) = 0.5874, *p* = 0.4460, prepulse intensity effect: *F* (3,71) = 9.789, *p* < 0.0001), followed by Bonferroni *post hoc* test: Kcnq3-W266L vs. WT, ns, not significant. Data are presented as mean ± S.E.M. **(G–H)** Immobility time in force swim assay. **(G)** Immobility time in force swim assay in male (*n* = 8 WT, n = 9 Kcnq3-W266L), Unpaired t-test (*t* = 1.620, *p* = 0.1275), ns, not significant. **(H)** Immobility time in force swim assay in female (*n* = 9 WT, *n* = 10 Kcnq3-W266L), Unpaired t-test (*t* = 0.6943, *p* = 0.4969), ns, not significant. Data are presented as mean ± S.E.M.

Utilizing a social interaction assay, we found that germline disruption of the Kcnq3 GABA binding site had no effect on sociability, as demonstrated by the longer amount of time spent with an unfamiliar mouse than an empty cup for both male and female Kcnq3-W266L mice (Figures 2A,B). In the prepulse inhibition assay, the Kcnq3-W266L mice displayed normal startle response, and normal prepulse inhibition to the three different startle stimuli (68, 71, and 77 dB), as well as the average prepulse in both male and female. This result indicates that the Kcnq3 GABA binding site is not involved in the sensorimotor gating system (Figures 2C–F). Furthermore, the absence of GABA binding sites on Kcnq3 had no effect on depression-like behaviors, as evidenced by the comparable immobility time in the forced swim test between the Kcnq3-W266L and wild-type mice (Figures 2G,H).

### The Kcnq3 GABA binding site is required specifically for spatial working memory in female mice

**Figure 3:**
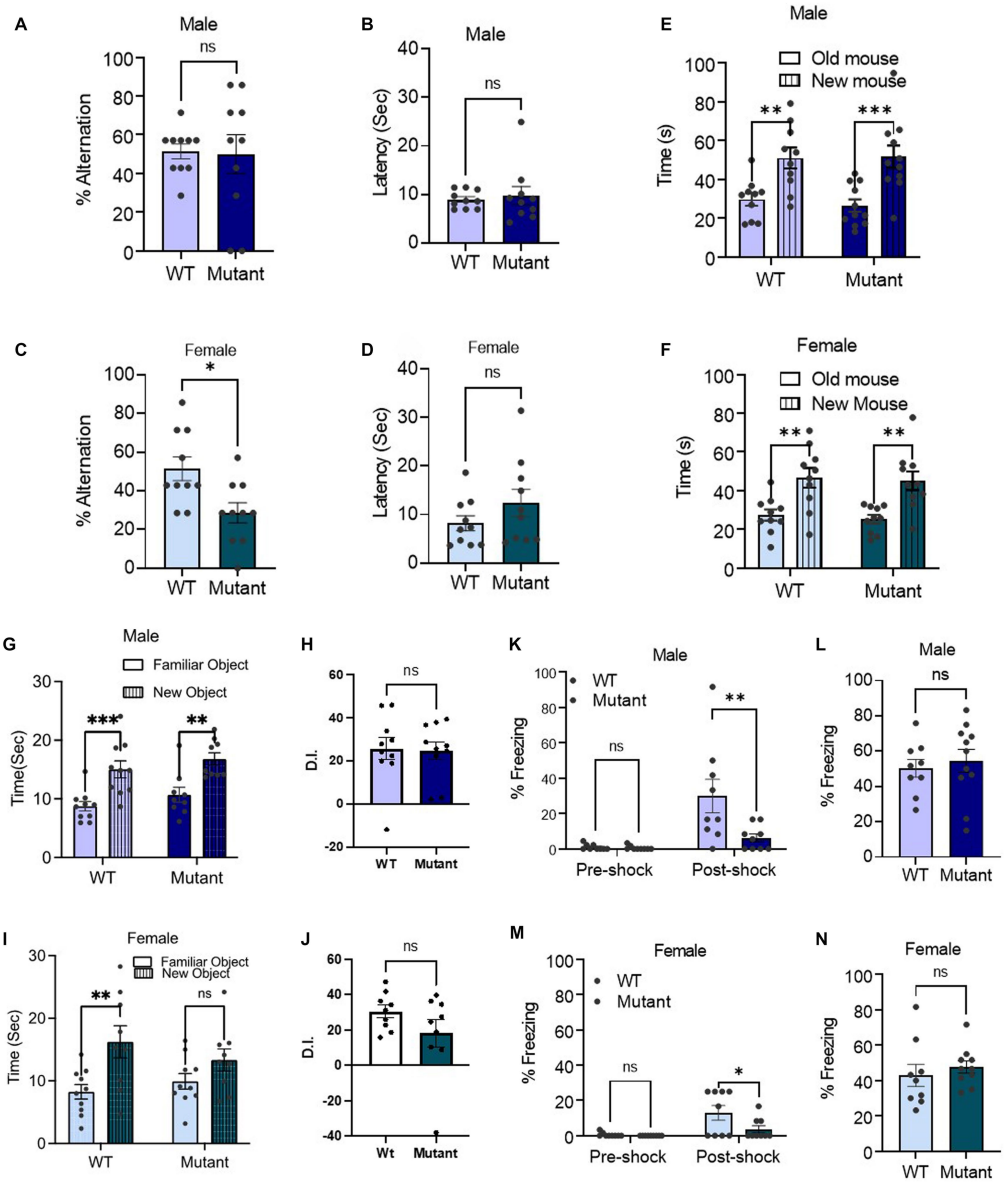
The KCNQ3 GABA binding site is required for spatial working memory in female mice but not affecting other memory. **(A–D)** Percent alternation and decision latency in *t*-maze assay. **(A)** Percent alternation in male (*n* = 10 WT, *n* = 10 Kcnq3-W266L), Unpaired *t*-test (*t* = 0.1330, *p* = 0.8957), ns, not significant. **(B)** Decision latency in male (*n* = 10 WT, *n* = 10 Kcnq3-W266L), Unpaired *t*-test (*t* = 0.4032, *p* = 0.6919), ns, not significant. **(C)** Percent alternation in female (*n* = 10 WT, *n* = 10 Kcnq3-W266L), Unpaired *t*-test (*t* = 2.848, *p* = 0.0107), WT vs. Kcnq3-W266L, ^*^p < 0.01. **(D)** Decision latency in male (*n* = 10 WT, *n* = 10 Kcnq3-W266L), Unpaired *t*-test (*t* = 1.307, *p* = 0.2075), ns, not significant. Data are presented as mean ± S.E.M. **(E–F)** Social novelty recognition. **(E)** In male (*n* = 10 WT, *n* = 11 Kcnq3-W266L), Two-way ANOVA (genotype effect: *F* (1,38) = 0.07334, *p* = 0.7880, novel mouse effect: *F* (1,38) = 25.62, *p* < 0.0001), followed by Bonferroni post-hoc test: old mouse vs. new mouse, ^**^p < 0.01, ^***^p < 0.001. **(F)** In female (*n* = 10 WT, *n* = 10 Kcnq3-W266L), Two-way ANOVA (genotype effect: *F* (1,36) = 0.2129, *p* = 0.6473, novel mouse effect: *F* (1,36) = 24.02, *p* < 0.0001), followed by Bonferroni *post hoc* test: old mouse vs. new mouse,^**^p < 0.01. Data are presented as mean ± S.E.M. **(G–H)** Novel object recognition in: **(G)** male (*n* = 10 WT, *n* = 9 Kcnq3-W266L), Two-way ANOVA (genotype effect: *F* (1,17) = 1.971, *p* = 0.1784, object effect: *F* (1,17) = 37.69, *p* < 0.0001), followed by Bonferroni post-hoc test: old object vs. new object, ^**^p < 0.01, ^***^p < 0.001; **(H)** Discrimination Index (D.I.) in male, unpaired *t*-test (*t* = 0.1332, *p* = 0.8955), Kcnq3-W266L vs. WT, ns, not significant. **(I)** female (n = 10 WT, *n* = 10 Kcnq3-W266L), Two-way ANOVA (genotype effect: *F* (1,36) = 0.2106, *p* = 0.6491, object effect: *F* (1,36) = 10.18, *p* = 0.0029), old object vs. new object, ^*^p < 0.05, ns, not significant. **(J)** D.I. in female, unpaired *t*-test (*t* = 1.429, *p* = 0.1721), Kcnq3-W266L vs. WT, ns, not significant. Data are presented as mean ± S.E.M. **(K–N)** Percent freezing in contextual fear conditioning. **(K)** Training day in male, (*n* = 9 WT, *n* = 9 Kcnq3-W266L), Two-way ANOVA (genotype effect: *F* (1,32) = 6.157, *p* = 0.0185, post-shock effect: *F* (1,32) = 12.57.24, *p* = 0.0012), followed by Bonferroni *post hoc* test: WT vs. Kcnq3-W266L, ^**^p < 0.01, ns, not significant; **(L)** Testing Day in male (*n* = 9 WT, *n* = 9 Kcnq3-W266L), unpaired *t*-test (*t* = 0.05024, *p* = 0.6215), WT vs. Kcnq3-W266L, ns, not significant. **(M)** Training day in female, (*n* = 9 WT, *n* = 10 Kcnq3-W266L), Two-way ANOVA (genotype effect: *F* (1,32) = 4.417, *p* = 0.0435, post-shock effect: *F* (1,32) = 11.91.24, *p* = 0.0016), followed by Bonferroni *post hoc* test: WT vs. Kcnq3-W266L, *p < 0.05; **(N)** Testing Day in female (*n* = 9 WT, *n* = 10 Kcnq3-W266L), unpaired *t*-test (*t* = 0.6767, *p* = 0.5077), WT vs. Kcnq3-W266L, ns, not significant. Data are presented as mean ± S.E.M.

We then assessed the impact of the Kcnq3-W266L mutation on spatial working memory, social recognition memory, object recognition memory and contextual long-term memory. In the T-maze spontaneous alternation test, which assesses spatial working memory, we found that female Kcnq3-W266L mice made more errors in alternating, indicating a decline in their spatial working memory impairment (Figures 3A–C). However, this was not observed in male Kcnq3-W266L mice. The latency of decision-making in the T-maze was unaffected by the Kcnq3-W266L mutation, in both male and female mice (Figures 3B–D).

Social recognition memory was intact in both male and female Kcnq3-W266L mice (Figures 3E,F), as evidenced by the significantly longer time spent with the novel mouse (unfamiliar) compared to the familiar mouse. In male and female mice, the object recognition memory was unaffected by the Kcnq3-W266L mutation, as demonstrated by spending more time with the novel object than the old object (Figures 3G–J). Similarly, the contextual long-term memory was also preserved in both male and female Kcnq3-W266L mice, as revealed by their similar freezing response on the testing day compared to wild-type mice (Figures 3K–N).

### Germline disruption of the Kcnq3 GABA binding site sex-dependently affects stress and pain

**Figure 4:**
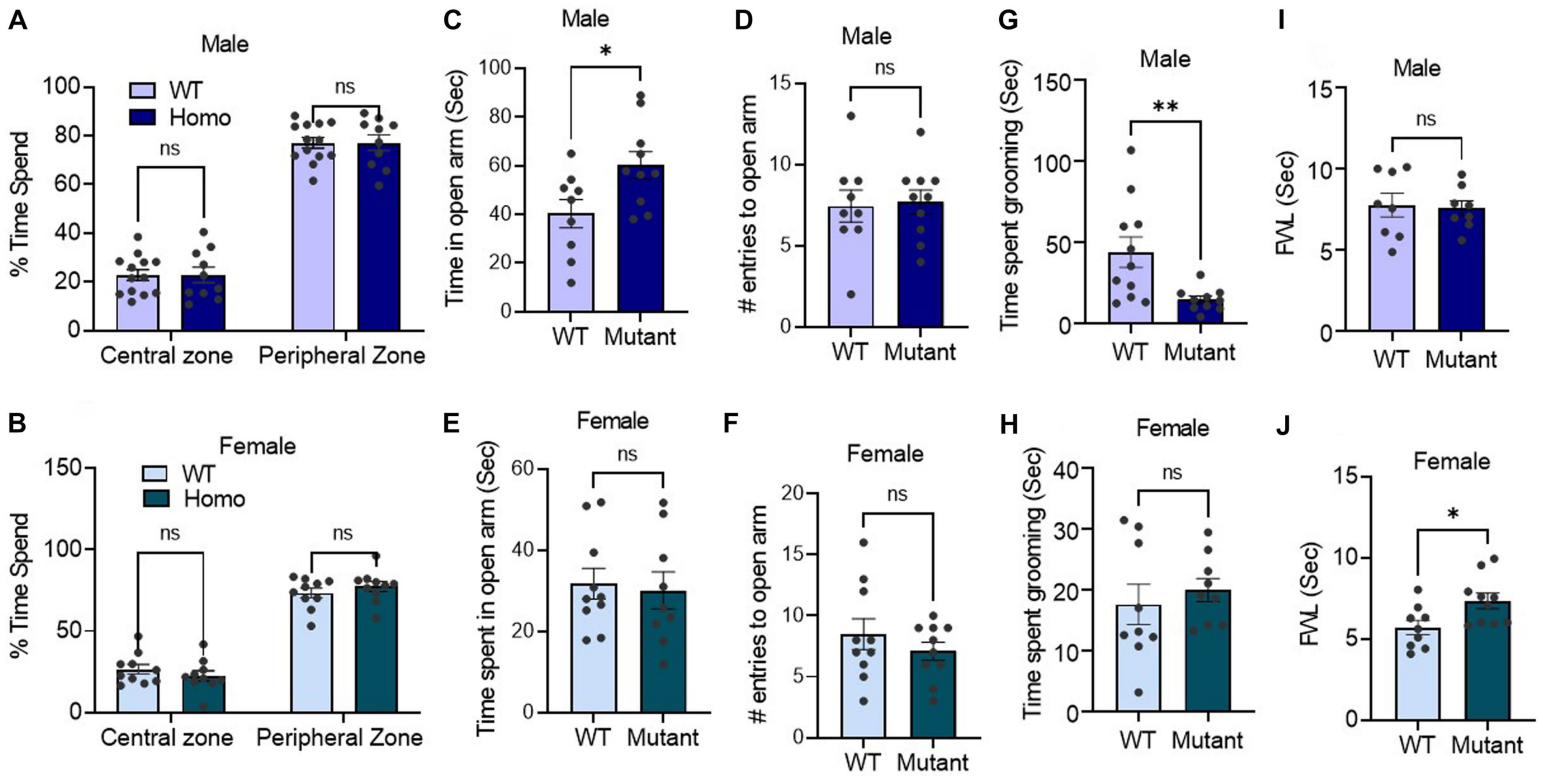
Germline disruption of the KCNQ3 GABA binding site alters stress and pain responses in a sex-dependent manner **(A,B)** Time spent in center vs. time in peripheral zone in open field assay. **(A)** In male (*n* = 13 WT, 10 Kcnq3-W266L), Two-way ANOVA (genotype effect: *F* (1,42) = 0.00, *p* > 0.999, zone effect: *F* (1,42) = 412.3, *p* < 0.0001), followed by Bonferroni *post hoc* test: WT vs. Kcnq3-W266L, ns, not significant. **(B)** In female (*n* = 10 WT, 10 Kcnq3-W266L), Two-way ANOVA (genotype effect: *F* (1,36) = 0.00, *p* > 0.999, zone effect: *F* (1,36) = 276.4, *p* < 0.0001) followed by Bonferroni *post hoc* test: WT vs. Kcnq3-W266L, ns, not significant. Data are presented as mean ± S.E.M. **(C–F)** Elevated-plus maze time spend and arm re-entrance. **(C–D)** Male (*n* = 9 WT, *n* = 10 Kcnq3-W266L): **(C)** time spent in open arms, unpaired t-test (*t* = 2.494, *p* = 0.0232), WT vs. Kcnq3-W266L, ^*^p < 0.05; **(D)** number of entries to the open arm, unpaired *t*-test (*t* = 0.9600, *p* = 0.3498), WT vs. Kcnq3-W266L, ns, not significant. (e-f) female (*n* = 10 WT, *n* = 9 Kcnq3-W266L): **(E)** time spent in open arms, unpaired t-test (*t* = 0.2893, *p* = 0.7758), WT vs. Kcnq3-W266L, ns, not significant. **(F)** number of entrances to the open arm in female, unpaired *t*-test (*t* = 0.8317, *p* = 0.4171), WT vs. Kcnq3-W266L, ns, not significant. Data are presented as mean ± S.E.M. **(G,H)** Time spent on grooming in: **(G)** male (*n* = 11 WT, *n* = 10 Kcnq3-W266L), unpaired t-test (*t* = 2.927, *p* = 0.0087), WT vs. Kcnq3-W266L, ^**^p < 0.01; (h) female (*n* = 9 WT, *n* = 9 Kcnq3-W266L), unpaired t-test (*t* = 0.6189, *p* = 0.5447), WT vs. Kcnq3-W266L, ns, not significant. **(I,J)** Foot withdraw latency in hotplate assay in: **(I)** male (*n* = 8 WT, *n* = 8 Kcnq3-W266L), Unpaired *t*-test (*t* = 0.2419, *p* = 0.8123), ns, not significant; **(J)** female (*n* = 9 WT, *n* = 10 Kcnq3-W266L), Unpaired t-test (*t* = 2.530, *p* = 0.0216), WT vs. Kcnq3-W266L, ^*^p < 0.05. Data are presented as mean ± S.E.M.

We next employed an open field test, elevated-plus maze assay, and self-grooming test to quantify stress-like behaviors. In the open field assay, which evaluates mouse aversion to open and brightly lit areas, male and female mutant and WT mice displayed similar behavior. Both groups spent more time in the peripheral zone than the central zone of the open field chamber during the initial 10 min of the experiment (Figures 4A,B).

Using the elevated plus maze, we measured the time spent in open and closed arms and entry count into open arms. Our findings indicated that the Kcnq3-W266L mice, both male and female, displayed similar entries into the open arms compared to the wild-type mice. However, the male Kcnq3-W266L mice spent a significantly longer time in the open arms, indicating a lower level of anxiety in these mice, while the female Kcnq3-W266L mice spent the same amount of time in the open arms as the wild-type mice (Figures 4C–E). This suggests that the male Kcnq3-W266L mice have a lower level of certain anxiety elements compared to the wild-type mice. Aligned with this, male Kcnq3-W266L mice exhibited reduced self-grooming time in a novel environment, providing evidence of decreased stress levels (Figure 4G). This effect was not observed in female Kcnq3-W266L mice.

On the other hand, female but not male Kcnq3-W266L mice exhibited an increased pain tolerance compared with the WT mice, evidenced by an increased foot withdrawal latency in the hotplate assay (Figures 4I,J). Lastly, while the results of the fear condition test revealed that the freezing response of the test day was comparable between WT and Kcnq3-W266L mice, it is noteworthy that the Kcnq3-W266L mice displayed a reduced freezing response on the training day, suggesting a decreased sensitivity to stress or pain in either sex (Figures 3K–M).

### Germline disruption of the Kcnq3 GABA binding site has sex- and brain region-dependent effects on neuronal activity

**Figure 5:**
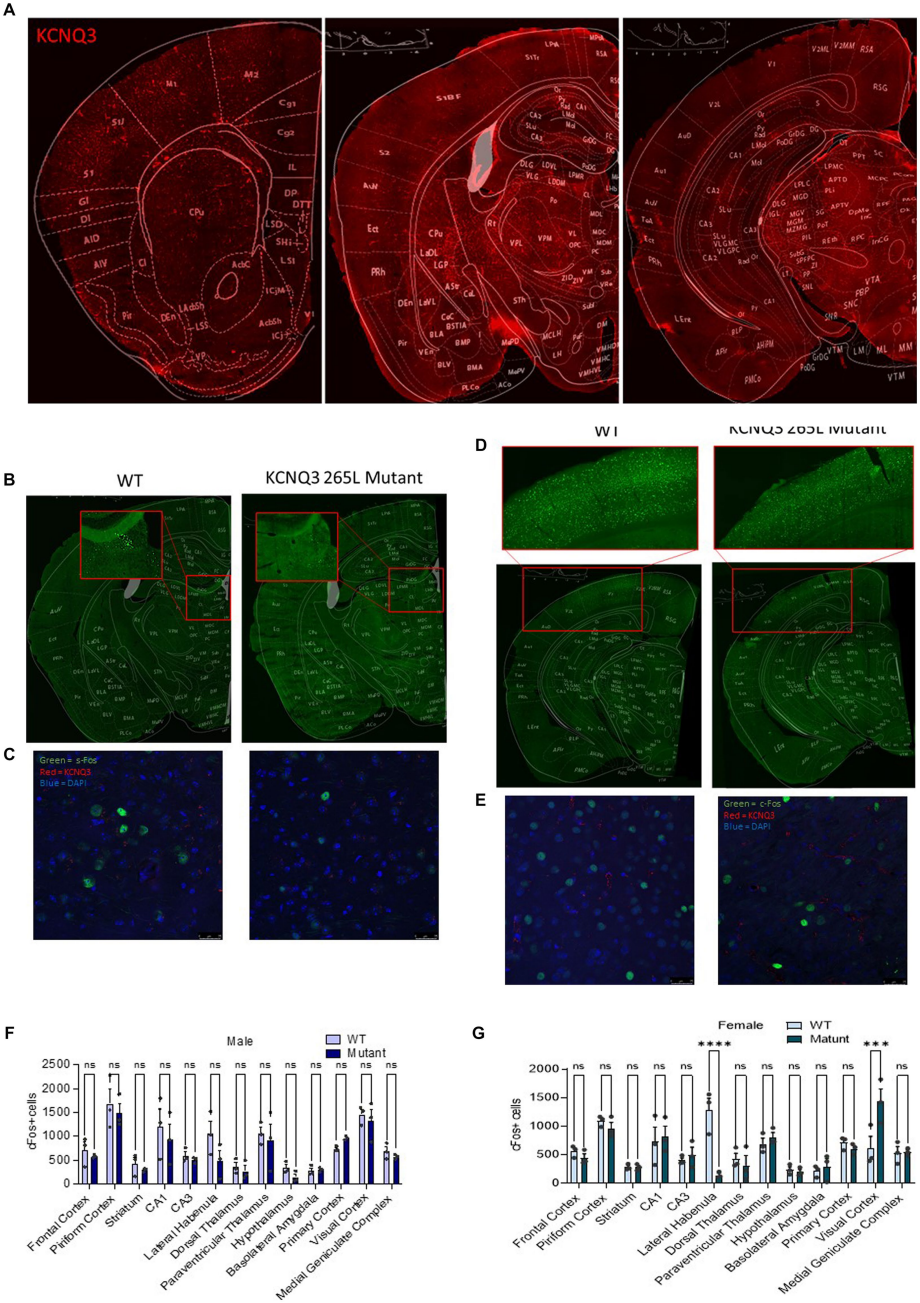
Germline disruption of the KCNQ3 GABA binding site causes regional changes in neuronal c-Fos expression. **(A)** Representative images showing KCNQ3 (red) staining in different regions of brain **(B)** Representative images showing c-Fos (green) staining in the lateral amygdala in WT and Kcnq3-W266L female mice **(C)** Representative images showing c-Fos (green), KCNQ3 (red), and DAPI (blue) staining in the lateral amygdala in WT and Kcnq3-W266L female mice, scale bar = 25 μm. **(D)** Representative images showing c-Fos (green) staining in the visual cortex in WT and Kcnq3-W266L female mice. **(E)** Representative images showing c-Fos (green), KCNQ3 (red), and DAPI (blue) staining in the lateral amygdala in WT and Kcnq3-W266L female mice, scale bar = 25 μm. **(F)** Quantitation of c-Fos positive cell density in the WT and Kcnq3-W266L male mice. Two-way ANOVA (genotype effect: *F* (1,52) = 3.873, *p* = 0.0544, brain regions effect: *F* (12,52) = 11.59, *p* < 0.0001, genotype x region interaction: *F* (12, 52) = 0.4952, *p* = 0.9082) followed by Bonferroni *post hoc* test: WT vs. mutant, ns, not significant. Data are presented as mean ± S.E.M. *n* = 3 sections from each of 3 mice per group. **(G)** Quantitation of c-Fos positive cell density in the WT and Kcnq3-W266L female mice. Two-way ANOVA (genotype effect: *F* (1,52) = 0.5017, *p* = 0.4819, brain regions effect: *F* (12,52) = 10.17, *p* < 0.0001, genotype x region interaction: *F* (12, 52) = 5.834, *p* < 0.0001) followed by Bonferroni *post hoc* test: WT vs. Kcnq3-W266L, ^***^p < 0.001, ^****^p < 0.0001, ns, not significant. Data are presented as mean ± S.E.M; *n* = 3 sections from each of 3 mice per group.

Along with the unique behavioral phenotype of the Kcnq3-W266L mice, our c-Fos immunoreactivity assay revealed alterations in neuronal activity in specific brain regions in the female Kcnq3-W266L mice (Figures 5A–G). The results showed a decrease in the number of c-Fos-positive cells in the lateral habenula and an increase in the visual cortex of the Kcnq3-W266L female mice (Figures 5B–E,G). Although there was a tendency towards a decrease in c-Fos-positive cells in the habenula of the Kcnq3-W266L male mice, this change was not statistically significant, and there were no other noticeable changes in neuronal activity in any other brain regions of the male Kcnq3-W266L mice (Figure 5F).

## Discussion

Our previous discovery of the direct activation of KCNQ3 and KCNQ5 channels by GABA, *via* a Trp-based binding site on the α subunit S5 segment (Manville et al., 2018) that was first discovered as a retigabine binding site (Schenzer et al., 2005), raised questions about its potential involvement in mediating GABA-regulated behavioral functions. To begin to shed light on these questions, we investigated the behavioral changes of mice with a mutation (W266L) at the GABA binding site of Kcnq3. GABA binding to KCNQ3, and to the KCNQ3 GABA binding site in KCNQ2/KCNQ3 heteromers (thought to be the predominant molecular correlate of the neuronal M-current), negative shifts the voltage dependence of activation, increasing channel activity at subthreshold potentials, reducing cellular excitability by hyperpolarizing the membrane potential (Manville et al., 2018). The W266L mutation prevents GABA binding to KCNQ3, with minimal effects on the baseline activity of KCNQ3 or KCNQ2/KCNQ3 channels; thus, the W266L mutation is a relatively specific way for uncovering physiologic effects of GABA binding to KCNQ3 without altering GABA interactions with other proteins, such as canonical GABA receptors (Manville et al., 2018). Here, we found that the Kcnq3-W266L mutation leads to distinctive and sex-specific behavioral phenotypes in mice, of which reduced nociceptive and stress responses were profound and sex-specific. Along with these behavioral changes, neuronal activity in brain regions known to be involved in nociception-stress pathways was altered.

In female mice, the Kcnq3-W266L mutation led to decreased nociceptive response, while in male mice, the mutation resulted in reduced stress responses. Notably, both male and female Kcnq3-W266L mice displayed reduced post-shock freezing behavior, known to reflect both fear and pain responses.

The pathways involved in regulating pain and stress/fear have been shown to have some overlap (Marcinkiewcz et al., 2009; Zheng et al., 2015; Nation et al., 2018; Sagalajev et al., 2018; Yin et al., 2020), and the GABAergic system has been implicated in both pain and stress/fear regulation (Lau and Vaughan, 2014; François et al., 2017; Hogri et al., 2022). For example, during fear conditioning, foot-shock sensory information is transmitted through pain pathways to the amygdala, a brain structure associated with fear processing (LEHNER et al., 2006; DeBerry et al., 2015). Therefore, our findings suggest that mutating the Kcnq3 GABA binding site in mice disrupts the overlapping pathways that regulate stress/fear and pain. This disturbance in stress/fear and pain pathways can manifest in a sex-dependent phenotype, presenting reduced nociceptive responses in females and reduced fear/anxiety in male mice. The results of our study highlight the complexity of GABAergic signaling and suggest that the GABA-KCNQ3 signaling pathway plays a selective role in modulating these functions in a sex-dependent and brain-region-dependent manner. Surprisingly, nearly 45% of both WT and mutant female mice did not freeze or exhibit other fear behaviors following the standard foot-shock intensity (0.7 mA). We would like to note that it is known that different strains can display variability in their fear response to foot-shock (Kazdoba et al., 2007). While our wild-type mice possess a C57BL/6 background, they were bred in-house from Kcnq3-W266L heterozygous mice, which might result in slightly different responses compared to the standard C57BL/6 background mice.

The exact mechanism and sites through which blocking the GABA binding site on KCNQ3 leads to changes in GABA activity is not fully understood and may involve multiple signaling pathways. Since the behavioral phenotypes observed in the Kcnq3-W266L mice may mimic those induced by GABA receptor activation, a simple explanation would be that blocking the GABA binding site on KCNQ3 causes a compensatory shift in GABA activity towards further activation of GABA receptors. However, this interpretation does not take into account the complexity of GABA roles in regulating neural circuits involved in movement, muscle tone, stress, and pain perception. For example, GABAergic neurons in the basal ganglia have dual effects on movement through depending on the specific nuclei involved and the balance of activity between the direct and indirect pathways. Increased GABA activity in the output regions of the basal ganglia (substantia nigra pars reticulata and medial globus pallidus) inhibits motor activity, whereas stimulating GABA neurons in the lateral globus pallidus increases motor activity (Yoshida, 1981; McGeer et al., 1984; BOLAM et al., 2000; Tisch et al., 2004; Hikosaka, 2007; Saunders et al., 2015; Tremblay et al., 2015). Similarly, GABAergic neurotransmission in the spinal cord, cortices, and brainstem can modulate pain in different ways. For example, GABA neurotransmission in the rostral agranular insular cortex can raise or lower the pain threshold, producing analgesia or hyperalgesia (Jasmin et al., 2003). On the other hand, KCNQ channels are necessary for normal mechanonociceptive responses in dorsal horn neurons receiving Aδ input (Passmore et al., 2012; CAI et al., 2015). Opening KCNQ2/3 channels directly with retigabine was shown to reduce pain (Blackburn-Munro and Jensen, 2003; Xu et al., 2010; Hayashi et al., 2014), and a gain-of-function mutation in *KCNQ3* has been shown to contribute to pain resilience (Yuan et al., 2021).

Although our current study did not determine the location in the brain where GABA induces effects through KCNQ3, the distribution of KCNQ3 and c-Fos results suggest that the habenula and visual cortex might be the primary sites in which GABA activation of KCNQ3-containing channels is of particular physiological importance, with this activation being sex-dependent.

The reduced c-Fos expression we observed in the habenula of Kcnq3-W266L female mice is a fascinating finding in light of previous studies showing that stressors can induce c-Fos expression in the lateral habenula (Wirtshafter et al., 1994). c-Fos is a well-known marker of neuronal activity and is frequently used to map brain activity, and thus, the observed reduction in c-Fos expression in the habenula of Kcnq3-W266L mice strongly suggests that habenular activity may be decreased in these animals. Furthermore, previous research has indicated that chronic stress exposure can decrease GABAergic inputs into lateral habenular neurons, which may be associated with altered nociceptive and fear responses (Shabel et al., 2014; Li et al., 2021; Lalive et al., 2022). Taken together with our current findings, which demonstrate decreased nociceptive and fear responses associated with reduced c-Fos expression in Kcnq3-W266L mice, these results suggest that GABAergic activation of KCNQ3 in the lateral habenula may play a role in the regulation of these responses.

The increase in c-Fos expression in the visual cortex of the Kcnq3-W266L female mice indicates increased neuronal activity in this region. The visual cortex is involved in processing visual information, and it also plays a role in the regulation of movement and spatial navigation. The activities of the three GABAergic interneurons (vasoactive intestinal peptide (Vip), somatostatin (SST neurons), and parvalbumin (PV neurons)) in the visual cortex are known to be diversely impacted by locomotion. For example, the activity of GABAergic-VIP neurons increases, whereas the activities of GABAergic-SST neurons increase or decrease during locomotion (Pfeffer et al., 2013; Polack et al., 2013; Fu et al., 2014; Reimer et al., 2014; Pakan et al., 2016; Urban-Ciecko and Barth, 2016; Dipoppa et al., 2018). Locomotion impact on GABAergic-PV neurons varies depending on the cortical depth (Dipoppa et al., 2018). On the other hand, PV and SST neurons activities have been linked with visual perception during an orientation discrimination task in T-maze (Song et al., 2020). Altogether, the decreased locomotion in the female mutated mice, as well as their declined spatial memory in the T-maze suggest that the increased activity in the visual cortex may be related to these behavioral changes. The role of GABA in these behaviors is complex, as it has multiple roles in regulating neural function, through regulating the excitability of neurons, and thus plays a role in shaping neuronal activity patterns to control the overall output of these neural circuits. Thus, decreased GABA activation of KCNQ3-containing channels could lead to inhibition or disinhibition of neuronal activity in the visual cortex. Although c-Fos is a well-established and widely used marker of neuronal activation, it is essential to highlight its limitations as a sole indicator of activity, given that c-Fos expression may not necessarily overlap with all KCNQ3 expressing neurons. Furthermore, there are other early immediate genes, such as Arc, Egr1, c-Jun, and Npas4, each with distinct expression patterns in different brain regions and neuronal types. Investigating the expression of multiple early immediate genes could offer a more comprehensive understanding of neuronal activation patterns and the underlying molecular mechanisms.

In conclusion, our study on the effects of a mutation at the GABA binding site of KCNQ3 on mouse behavior has revealed complex and nuanced results. The mutation leads to behavioral alterations in a sex-dependent manner, including decreased spontaneous motor activity, spatial memory, and nociceptive response in female mice, as well as altered post-shock freezing behavior in male and female mice. The results suggest that the GABA-KCNQ3 signaling pathway plays a selective role in modulating fear, pain, and emotional processing in a manner that is dependent on both sex and brain region. It is also important to note that KCNQ3-W266 is also required for binding of, and activation by, other endogenous ligands, including the ketone body β-hydroxybutyric acid, which activates KCNQ3 with similar potency and efficacy to that of GABA (Manville et al., 2020), and γ-amino-β-hydroxybutyric acid, which acts as a high-affinity partial agonist of KCNQ3 (when compared to GABA) (Manville et al., 2018). Further studies are necessary to fully understand the exact mechanisms behind the behavioral and neuronal activity changes observed upon disruption of the KCNQ3 GABA binding site and to assess their potential therapeutic implications.

## Data availability statement

The raw data supporting the conclusions of this article will be made available by the authors, without undue reservation.

## Author contributions

AA and GA conceived the experimental design and wrote the manuscript. RY contributed to experiment design and mouse genotyping. KC performed behavioral and immunostaining experiments. CE and TT assisted with data analysis. OC helped with experiment design and manuscript writing. All authors contributed to the article and approved the submitted version.

## Funding

We are grateful for financial support from the US National Institutes of Health, National Institute of Neurological Disorders and Stroke (NS107671 to G.W.A.). The authors acknowledge the support of the Chao Family Comprehensive Cancer Center Transgenic Mouse Facility Shared Resource, supported by the National Cancer Institute of the National Institutes of Health under award number P30CA062203. The content is solely the responsibility of the authors and does not necessarily represent the official views of the National Institutes of Health.

## Conflict of interest

The authors declare that the research was conducted in the absence of any commercial or financial relationships that could be construed as a potential conflict of interest.

## Publisher’s note

All claims expressed in this article are solely those of the authors and do not necessarily represent those of their affiliated organizations, or those of the publisher, the editors and the reviewers. Any product that may be evaluated in this article, or claim that may be made by its manufacturer, is not guaranteed or endorsed by the publisher.
